# Enhancement of Antitumor Immunity Using a DNA-Based Replicon Vaccine Derived from Semliki Forest Virus

**DOI:** 10.1371/journal.pone.0090551

**Published:** 2014-03-07

**Authors:** Liang Zhang, Yue Wang, Yi Xiao, Yu Wang, JinKai Dong, Kun Gao, Yan Gao, Xi Wang, Wei Zhang, YuanJi Xu, JinQi Yan, JiYun Yu

**Affiliations:** 1 Beijing Institute of Basic Medical Sciences, Haidian district, Beijing, China; 2 National Center for AIDS/STD Control and Prevention, China-CDC, Beijing, China; 3 Department of Urology, First Affiliated Hospital of General Hospital of PLA, Beijing, China; University Paris Sud, France

## Abstract

A DNA-based replicon vaccine derived from Semliki Forest virus, PSVK-shFcG-GM/B7.1 (**Fig. 1a**) was designed for tumor immunotherapy as previously constructed. The expression of the fusion tumor antigen (survivin and hCGβ-CTP37) and adjuvant molecular protein (Granulocyte-Macrophage Colony-Stimulating Factor/ GM-CSF/B7.1) genes was confirmed by Immunofluorescence assay in vitro, and immunohistochemistry assay in vivo. In this paper, the immunological effect of this vaccine was determined using immunological assays as well as animal models. The results showed that this DNA vaccine induced both humoral and cellular immune responses in C57BL/6 mice after immunization, as evaluated by the ratio of CD4^+^/CD8^+^ cells and the release of IFN-γ. Furthermore, the vaccination of C57BL/6 mice with PSVK-shFcG-GM/B7.1 significantly delayed the in vivo growth of tumors in animal models (survivin^+^ and hCGβ^+^ murine melanoma, B16) when compared to vaccination with the empty vector or the other control constructs (**Fig. 1b**). These data indicate that this type of replicative DNA vaccine could be developed as a promising approach for tumor immunotherapy. Meanwhile, these results provide a basis for further study in vaccine pharmacodynamics and pharmacology, and lay a solid foundation for clinical application.

## Introduction

Over the past few years, tremendous progress has been achieved in tumor therapy by using antigen-encoded plasmid DNA as a vaccine. In comparison to recombinant subunits and inactivated vaccines, DNA vaccines are relatively simple and inexpensive to produce, and induce longer lasting immune responses [1]. In addition, it has been demonstrated that DNA vaccination can induce both CD4^+^ (Th1) and CD8^+^ cytotoxic T lymphocyte (Tc) immune responses [2]. Therefore, recombinant DNA vaccines have certain advantages and open a new avenue for cancer therapy.

In recent years, a vector system, which is based on RNA virus replication components and has a "self-replication" function, has been developed. Alphaviruses are single-stranded RNA (ss-RNA) viruses with positive polarity [3]. They have an envelope consisting of two or three major proteins that form heterologous spikes. Among the many alphaviruses, Semliki Forest virus (SFV) [4], Sindbis virus (SIN) [5] and Venezuelan equine encephalitis (VEE) virus [6] have been engineered as efficient delivery and expression vectors. The layered DNA-RNA vector system is one of three types of replicative vectors. The SP6 RNA polymerase promoter has been replaced by a CMV promoter, which allows the direct application of the vector as a transfection agent. Due to the presence of the SFV replicase genes, extensive RNA replication will result in superior gene expression compared to conventional plasmid vectors. Self-replication and transcription of the replicon DNA vaccine occurs in the cytoplasm and eventually eliminates the risk of integration into the host cell genome and greatly improves the safety [7]. Members of this family have served as a basis for viral vector and DNA plasmid vaccines for infectious diseases and cancer [8]. The application of alphavirus vectors as vaccines has included the administration of SFV, SIN and VEE virus as layered DNA-RNA vectors [9], [10], [11], [12], [13]. The most popular approach has been the intratumoral injection of alphavirus vectors which carry reporter and/or therapeutic genes. For instance, SFV vectors expressing the p40 and p35 subunits of IL-12 resulted in significant tumor regression and inhibition of tumor blood vessel formation in a murine melanoma cell (B16 cell) tumor model [14]. In another application, the expression of the murine VEGFR-2 from SFV vectors led to the inhibition of angiogenesis, which reduced tumor growth and metastasis in mice [15].

Regarding target gene selection, survivin could be an ideal molecule because it is ubiquitously expressed in embryonic tissues and tumor cells, but not in normal tissues [16], [17]. It is reported that survivin-targeting therapy can induce apoptosis in tumor cells but has no effect on normal tissues [18], [19], [20]. In addition, human chorionic gonadotropin (hCG), which is ubiquitously expressed in almost all tumor cells, is also an ideal candidate for DNA vaccines [21].

hCG is composed of a heterodimer of an alpha and a beta subunit, and the hCGβ single chain or the hCGβ core fragment (hCGβ-CTP37) can be selectively secreted by many tumor cells. Given that hCG is related to tumor metastasis and immunological tolerance [22], [23], [24], hCGβ-based immunological therapy has been developed and is currently on track for clinical trials to prevent the recurrence and metastasis of tumors after operation in pancreas and colorectal cancers [22].

Immunological tolerance elicited by homogeneity is also a major problem in immune therapy. To circumvent this phenomenon, we plan to use a chimeric gene that expresses heterogeneity of tumor-associated antigen. To further improve immunogenicity, we employed molecules that facilitate the recognition of Antigen presenting cells (APCs) and cell proliferation. The positive association between the molecular adjuvants and immune efficiency was highlighted by previous studies as follows: B7 binding to its ligand, CD28, can promote T cell proliferation and the secretion of chemokines [25], [26]; GM-CSF can activate dendritic cells as well as promote the proliferation of Th cells and effector cells [27], [28], [29]; Immunoglobulin G Fc fragment (IgG Fc) is involved in antibody-dependent immunomodulatory and cytotoxic functions [30], [31]; and the membrane anchor signal peptide of Glycosylphosphatidylinositol(GPI) can anchor the fusion protein to the cell membrane and facilitate the recognition of APCs [32], [33], [34].

Based on these findings and to improve the efficacy of anti-tumor therapeutic vaccines, we constructed a recombinant vaccine based on SFV. The expression of the fusion tumor antigen and adjuvant molecular protein gene was confirmed in vitro and in vivo [35]. We then studied its antitumor efficacy and immunological mechanisms in vitro and in vivo to provide the groundwork for developing an anti-tumor therapeutic vaccine.

## Material and Methods

### Ethics statement

Pathogen-free, female C57BL/6 mice were obtained from the Beijing Experimental Animal Center. The animals used in this study were raised to 6-8 weeks old and were maintained in accordance with the Guide for the Care and Use of Laboratory Animals (NIH Publication No. 85-23, Revised 1996). Experimental procedures were in strict agreement with international guidelines for the care and use of laboratory animals and approved by Animal Ethics Committee of Institute of Basic Medicine Sciences. We observed animal ethics during the research by complying with 3R principles (Replacement, Reduction, and Refinement).

### Target cell lines and reagents

The C57BL/6 melanoma cell line B16F10 (ATCC CRL-6475) was kindly provided by my colleague Dr. Jia Zou (Beijing Institute of Radiation Medicine), which was purchased from the American Type Culture Collection (ATCC). All cells were cultured in RPMI-1640 medium (Gibco-BRL, Gaithersburg, MD, USA) supplemented with 10% fetal calf serum, 100 units/mL penicillin, and 100 µg/mL streptomycin (all purchased from Life Technologies, Corporation. Grand Island, NY, USA). Cells were incubated at 37°C in an atmosphere containing 5.0% CO2 and saturating humidity. The medium was changed every 2–3 days. The full-length human survivin and hCGβ cDNA fragments were amplified by PCR and inserted into the eukaryotic expression vector pIRES-neo. After identification by restriction digestion and PCR, the recombinant plasmids pIRES-neo-SUR-(his)_6_ and pIRES-neo-hCGβ-(his)_6_ were obtained. These plasmids were transfected into B16F10 cells using Lipofectamine 2000. After selection using G418, the resulting target cells were designated B16F10-SUR and B16F10-hCGβ. The transfected cells were maintained in medium supplemented with 1 mg/mL G418 (Geneticin, Invitrogen, Carlsbad, CA, USA).

### Peptides

Human survivin, MHC class I H2-K^b^ restricted peptides (a.a. 80–88, AYACNTSTL) and human hCGβ, MHC class I H2-K^b^ restricted peptides (a.a. 109–118, TCDDPRFQDS) were purchased from SBS Genetech Co. Ltd., Beijing, China. Purity of >95% was confirmed by high-performance liquid chromatography (HPLC) and mass spectrometry (MS). Peptides were dissolved at 1 mg/mL in DMSO (Sigma Chemical, SAINT LOUIS, MO, USA), aliquoted in a small volume, and stored at –70°C until further use.

### Characterization of transfected target cells

B16F10 cells that stably expressed human survivin and hCGβ were harvested and lysed in phosphate-buffered saline (PBS) buffer containing 2 µg/ml aprotinin, 100 µg/ml phenylmethylsulfonyl fluoride, 2 µg/ml leupeptin and 1% Nonidet P-40. The proteins were separated by 12% Sodium dodecyl sulfate (SDS)-PAGE then were transferred onto a nitrocellulose membrane (Bio-Rad Laboratories, Hercules, CA, USA). The membranes were blocked by incubation in 5% nonfat dried milk then washed and incubated with a mouse anti-his tag antibody (Zhongshan Golden Bridge Biotechnology Co. Ltd., Beijing,China). Subsequently, the membranes were incubated with a horseradish peroxidase-labeled goat anti-mouse IgG (Zhongshan Golden Bridge Biotechnology Co. Ltd., Beijing, China). The membrane was visualized using the super ECL Plus Western blotting system (Applygen Technologies Inc., Beijing, China).

### Vaccination and analysis of antitumor activity in vivo

For the in vivo tumor prevention experiments, female C57BL/6 mice (n = 5) were immunized with 50 µg/100 µl PSVK, PSVK-GM/B7.1, PSVK-shFcG, or PSVK-shFcG-GM/B7.1 plasmid via intramuscular injection and electric pulsing then boosted twice at intervals of 10 days. As a control, mice were vaccinated with 100 µl PBS. Ten days after the last immunization, the mice were inoculated in the left flank with 1×10^5^ B16F10-SUR or B16F10-hCGβ cells resuspended in 100 µl PBS. The tumors were monitored every 2 days, and the tumor dimensions were determined by measurement with calipers (length and width). The values were inserted into the following formula: tumor volume (mm^3^)  =  0.5 × (length × width^2^). Survival was followed until 90 days after the tumor challenge.

For the in vivo therapeutic experiments, the mice received a single vaccination 10 days before tumor cell challenge, and the female C57BL/6 mice (n = 5) were injected with 1×10^5^ B16F10-SUR or B16F10-hCGβ cells/mouse on day 0 subsequently, the mice were immunized with the DNA vaccine via intramuscular injection and electric pulse on days 1, 8 and 15. Tumor development was monitored in individual mice every 2 days, and the tumor size was calculated using the formula described above. Survival was followed until 60 days after the tumor challenge.

### Antibody assay

Human survivin and hCGβ-CTP37-specific antibodies were detected in the sera of the immunized mice using Enzyme-linked immunosorbent assay (ELISA). 14, 28 days after the final vaccination, the antibody titers in the sera of vaccinated mice were detected. Purified human survivin and hCGβ-CTP37 protein was precoated into 96-well plates at 0.25 µg/well; the vaccinated mouse serum was obtained from the fossa orbitalis veins. The sera was added into the plate with precoated proteins and co-cultured at 4°C for almost 12 h. After washing with PBST (0.05% Tween 20 in PBS), the plates were blocked with 1% Bovine serum albumin (200 µl/well) for 1 h at 37°C followed by the addition of sera from the different vaccinated mice and incubation for 2 h at 37°C. Horse radish peroxidase (HRP)-labeled goat anti-mouse antibody (Sigma, St. Louis, MO, USA) was added, and then the plate was incubated with Tetramethyl benzidine (TMB) solution. The OD values were measured using an ELISA reader (Bio-Rad Laboratories Inc. Hercules, CA, USA).

### Cytotoxicity assay

The Cytotoxic T lymphocyte (CTLs) against target cells was detected using a nonradioactive lactate dehydrogenase (LDH) release assay (CytoTox96; Promega, Madison, WI, USA). Five groups of female C57BL/6 mice (n = 3) were challenged with B16F10-survivin or B16F10-hCGβ cells followed by vaccination with 50 µg of PSVK-shFcG-GM/B7.1, PSVK-shFcG, PSVK-GM/B7.1, or PSVK vectors via intramuscular injection and electric pulsing on day 1 or left unimmunized (negative control). These mice were boosted twice with the same regimen as the first vaccination on days 8 and 15. Tumor-bearing mice were euthanized 14 days after the last immunization, and splenocytes from immunized mice or control mice were used as effector cells in a nonradioactive cytolytic analysis. Splenocytes from immunized or control groups mice were plated in round-bottom 96-well plates, and were stimulated with H2-K^b^ class I restricted peptides for human survivin (a.a. 80–88, AYACNTSTL) or H2-D^b^ class I restricted peptides for human hCGβ (a.a. 109–118, TCDDPRFQDS) for 4h. After this process, the effector cells were added to the target cells (B16F10-SUR or B16F10-hCGβ) in a 96-well plate at effector. Target Effector/target cell ratio (E:T) ratios of 40:1, 20∶1 and 10∶1 (tested in triplicate). The procedure was performed according to the manufacturer’s instructions. Cytotoxicity was calculated using the following formula: cytotoxicity %  =  (E-Se-St)/(Mt-St)×100%; where E is the experimental LDH release in effector plus target cell co-cultures, Se is the spontaneous release by effector cells alone, St is the spontaneous release by target cells alone and Mt is the maximal release by target cells.

### Measurement of IFN-γ secretion using an ELISPOT assay

Peptide-specific T cells from vaccinated mice were counted by IFN-γ enzyme-linked immunospot (ELISPOT). The spleens of immunized mice were collected 14 days after the final DNA injection and were suspended in RPMI 1640 supplemented with 10% FCS, 2 mmol/L L-glutamine, 100 units/ml penicillin, and 100 µg/ml streptomycin. A total of 4×10^5^ spleen cells were added to each well of a 96-well plate and were stimulated with synthetic peptide at 1 µg/µl. The well was precoated with 2.5 µg/mL rat anti-mouse IFN-γ (Dakewe Biotech Ltd, Shenzhen, China). Splenocytes from unimmunized mice were used as a control. After 36 h of incubation, the cells were removed, and biotinylated rat anti-mouse- Interferonγ(IFNγ) (Dakewe Biotech Ltd., Shenzhen, China) was added. The plates were incubated for another 1 h at 37°C then washed to remove unbound antibody. Bound antibody was detected by incubating the plates with avidin-HRP (Dakewe Biotech Ltd, Shenzhen, China) for 1 h at 37°C. The substrate 3-amino-9-ethylcarbazole (AEC) was added at 100 µl per well, and the plate was incubated for 45 min. The AEC solution was discarded, and the plates were washed six times with water. The visualized cytokine spots were enumerated using the ImmunoSpot analyzer (CTL), and the results were expressed as the number of cytokine-producing cells per 4×10^5^ cells. A Wilcoxon two-tail rank test was performed to determine whether there was a statistically significant difference between the numbers of IFNγ secreting cells in the wells stimulated with the different vaccines.

### Isolation of tumor-infiltrating lymphocytes (TILs) and flow cytometry analysis

Individual melanoma tumors (0.1 g) were dissociated with 5 ml of 0.1% dispase (Roche, Germany) for 30 min at 37°C. The dissociated cells were removed by gentle aspiration and were placed into an equivalent volume of RPMI 1640. Fresh, pre-warmed enzyme solution was added again to the partially dissociated tumor, and this procedure was repeated twice. The cells were washed twice with 1 ml PBS and were sieved repeatedly to remove tissue fragments and debris, yielding a homogeneous cell suspension. Cells were collected by centrifugation and incubated in staining medium containing fluorochrome-conjugated antibodies (Fluorescein isothiocyanate-anti-CD4, PE-anti-CD8; BD, USA) for 40 min at 4°C (antibodies were used at 2 µg/ml). Subsequently, the cells were washed and analyzed using a flow cytometer to determine the percentage of CD4^+^ and CD8^+^ T lymphocytes.

### Statistical analysis

A statistical analysis was performed using commercially available software (SPSS 17.0). To compare individual time-points, a one-way ANOVA was used to compare three or more groups. Student’s t-test was used to compare the means between two groups. Survival curves were compared by the log-rank test. Survival time was calculated by the Kaplan-Meier method. Differences for which p <0.05 were considered statistically significant.

## Results

### Characterization of transfected target cells

**Figure 1:**
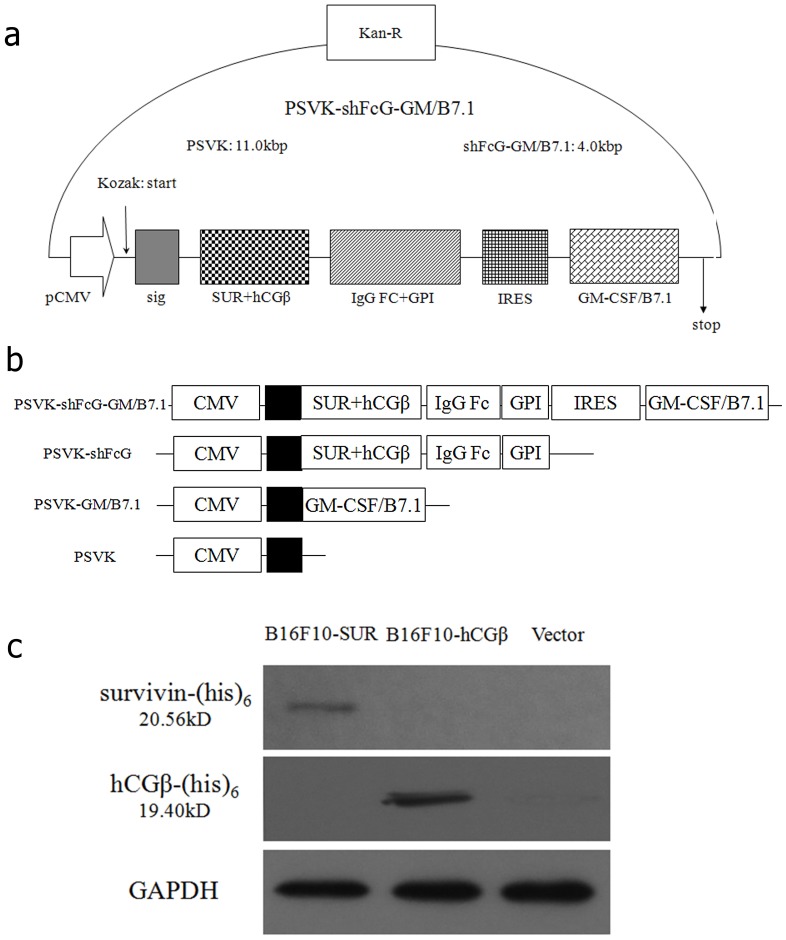
Design of the vaccine and the expression of tumor antigen in target cells. (a): Design of the DNA-based replicon anti-tumor vaccine PSVK-shFcG-GM/B7.1. (b): Schematic representation of expression vectors. The shFcG-GM/B7.1 fusion gene, the shFcG fusion gene, and the GM/B7.1 fusion gene with a signal sequence were cloned into the PSVK vector under the control of the CMV promoter. ▪indicates signal sequences. (c): Expression of survivin and hCGβ in target tumor cells (B16F10-SUR and B16F10-hCGβ). Murine B16F10 cells were transfected with recombinant vectors containing pIRES-neo-SUR, pIRES-neo-hCGβ or the control vector.

The expression of the survivin and hCGβ proteins in the target tumor cells (B16F10-SUR and B16F10- hCGβ) was analyzed by Western blotting (Fig. 1c). The survivin-(his)6 and hCGβ-(his)6 fusion proteins were successfully detected using a mouse anti-his tag antibody in the B16F10 cells transfected with pIRES-neo-SUR and pIRES-neo-hCGβ, respectively (**Fig. 1c**).

### Induction of antitumor immunity

**Figure 2:**
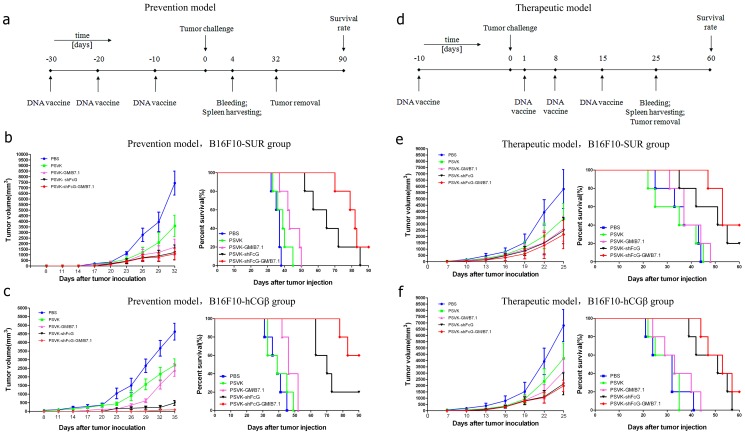
Anti-melanoma immune response induced by the anti-tumor DNA vaccine PSVK-shFcG-GM/B7.1 in two immunization strategies. (a) Schematic illustration of prophylactic vaccination. Three vaccinations with 50 µg plasmid DNA were applied at 10-day intervals in C57BL/6 mice (n = 15). Tumor cell (1×105 B16-SUR-(his)6 or B16-hCGβ-(his)6) injection, bleeding, spleen harvesting, tumor removal, and determination of survival rate were performed at the time-points indicated. (b and c) Tumor volume (mm3) in mice treated with PSVK-shFcG-GM/B7.1 and controls. The survival rate of the PSVK-shFcG-GM/B7.1-treated mice was 20% on day 90 for B16F10-SUR and 60% on day 90 for B16F10-hCGβ melanoma cells. (d) Therapeutic vaccination scheme. Primary tumors were induced by injecting 1×105 B16-SUR-(his)6 or B16-hCGβ-(his)6 cells (n = 15) followed by therapeutic vaccination with 50 µg plasmid DNA starting on day 1 of the primary tumor challenge. Bleeding, spleen harvesting, tumor removal, and the determination of survival rate were performed at the time-points indicated. (e and f) Tumor volume (mm3) in the mice treated with PSVK-shFcG-GM/B7.1 and controls is shown. The survival rate of the PSVK-shFcG-GM/B7.1-treated mice was 40% on day 60 for B16F10-SUR and 20% on day 60 for B16F10-hCGβ melanoma cells.

To investigate protective antitumor immunity, we used two tumor cell models and two immunization strategies. The tumors grew progressively in immunized mice, but there was an apparent protection from tumor growth in mice immunized with PSVK-shFcG-GM/B7.1 (**Fig. 2a-c**). The results are expressed as the mean tumor volume ± SD (one-way ANOVA, p<0.01). The survival of the mice treated with PSVK-shFcG-GM/B7.1 was also significantly greater than the other groups’ immunized mice (p <0.01 by the log-rank test).

The therapeutic efficacy of the DNA vaccine encoding shFcG-GM/B7.1 was next tested in established tumors. The mice vaccinated with PSVK-shFcG-GM/B7.1 demonstrated the lowest average tumor volume on day 25 compared with the mice vaccinated with the other constructs (one-way ANOVA, P<0.01; the data are expressed as the mean tumor volume ± SD, **Fig.** **2d-e**). These data clearly show that therapeutic vaccination significantly delayed tumor growth when compared with the controls. Furthermore, the survival of the PSVK-shFcG-GM/B7.1 treated mice was significantly longer than that of any other immunized groups (p<0.01 by log-rank test). In summary, these results show that vaccination with PSVK-shFcG-GM/B7.1 induces a strong antitumor response in a mouse tumor model, which in turn may contribute to greatly reducing tumor growth and significantly prolonging the longevity of tumor-bearing mice.

### Antibody assays

**Figure 3:**
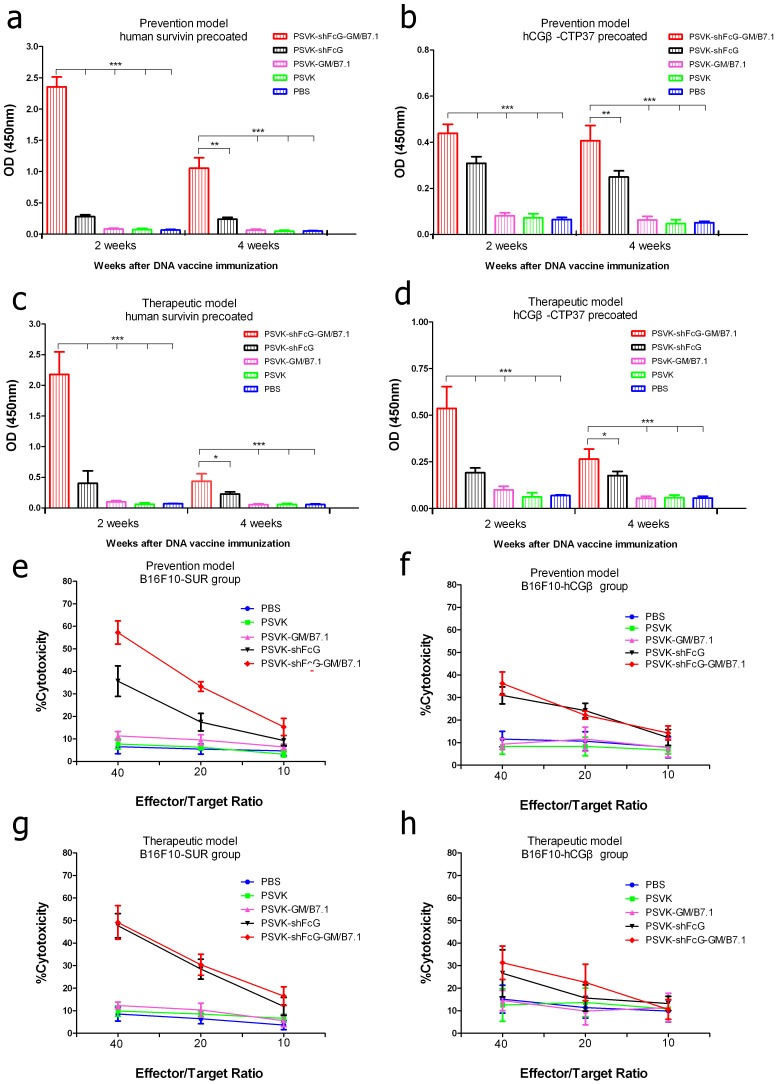
Induction of specific antibody responses and cytotoxic responses. Specific antibody responses: Mice were immunized with different DNA constructs, and sera were collected at 2 weeks and 4 weeks after DNA immunization. The antigen-specific antibodies from sera of the different mouse groups were determined at a 1∶100 dilution by ELISA. (a) Prevention model, Pre-coated with recombinant survivin protein; (b) Prevention model, Pre-coated with recombinant hCGβ-CTP37 protein; (c) Therapeutic model, Pre-coated with recombinant survivin protein; (d) Therapeutic model, Pre-coated with recombinant hCGβ-CTP37 protein. Cytotoxic responses: Splenocytes were removed from tumor-bearing mice 2 weeks after the final immunization. The splenocytes were co-cultured for 4 h with the target cells (B16F10-SUR or B16F10- hCGβ). The percent target cell killing by the splenocytes from differently immunized mice is shown. (e and f) Prevention model; (g and h) Therapeutic model. (e and g) Splenocytes from the mice in the prevention model were the effectors, and B16F10-SUR cells were the target cells. (f and h) Splenocytes from mice in the therapeutic model were the effectors, and B16F10- hCG cells were the target cells.

Specific antibodies also have a role in antitumor immunity. We used ELISA to measure antigen-specific antibody titers in the sera of immunized, tumor-bearing mice (both immunization strategies); **Fig. 3a-b**: prevention model; **Fig. 3c-d**: therapeutic model). Relatively higher antibody titers were detected in the sera of PSVK-shFcG-GM/B7.1-immunized mice compared to mice immunized with the other constructs (p<0.001 by one-way ANOVA). The PSVK-shFcG-immunized group also exhibited a higher titer of antigen-specific antibodies. As indicated in **Table 1**, the antibody titers in the different groups from high to low were PSVK-shFcG-GM/B7.1, PSVK-shFcG, PSVK-GM/B7.1 and control.

**Table 1:** Antibody titers in the groups of mice vaccinated with the different recombinant constructs.

Groups		human survivin pre-coated		human hCGβ-CTP37 pre-coated	
		2 weeks	4 weeks	2 weeks	4 weeks
prevention model	PBS	<1∶50	<1∶50	<1∶50	<1∶50
	PSVK	<1∶50	<1∶50	<1∶50	<1∶50
	PSVK- GM/B7.1	<1∶50	<1∶50	<1∶50	<1∶50
	PSVK- shFcG	1∶400	1∶200	1∶400	1∶200
	PSVK-shFcG-GM/B7.1	1∶12800***	1∶3200***	1∶800***	1∶800***
therapeutic model	PBS	<1∶50	<1∶50	<1∶50	<1∶50
	PSVK	<1∶50	<1∶50	<1∶50	<1∶50
	PSVK -GM/B7.1	<1∶50	<1∶50	<1∶50	<1∶50
	PSVK- shFcG	1∶3200	1∶200	1∶400	1∶200
	PSVK-shFcG-GM/B7.1	1∶102400***	1∶800***	1∶1600***	1∶800***

*** Significant differences as indicated by a statistical analysis of p<0.001.

### Cytotoxicity assay

To determine whether immunization with PSVK-shFcG-GM/B7.1 induces strong CTL responses in mice, we performed a nonradioactive LDH release assay. The results showed that splenocytes from mice immunized with PSVK-shFcG-GM/B7.1 killed B16F10-SUR and B16F10-hCGβ target cells much more efficiently than splenocytes from mice immunized with other vectors (both immunization strategies; **Fig. 3e-f**: prevention model; **Fig. 3g-h**: therapeutic model).

In the prevention experiments, effector cells of the PSVK-shFcG-GM/B7.1 group showed a percent cytotoxicity of 57.25% against B16F10-SUR target cells when the E:T ratio was 40:1, 33.27% when the ratio was 20:1, and 15.31% when the ratio was 10:1. Using B16F10-hCGβ cells as the target, the cytotoxicity of effector cells was 36.25%, 22.15% and 14.27% for E: T ratios of 40:1, 20:1, and 10:1, respectively.

In the therapeutic experiments, effector cells of the PSVK-shFcG-GM/B7.1 group showed a cytotoxicity of 49.16% against B16F10-SUR target cells when the E:T ratio was 40∶1, 30.39% when the ratio was 20∶1, and 16.48% when the ratio was 10∶1. Using B16F10-hCGβ as the target, the cytotoxicity of effector cells was 31.29%, 22.56% and 10.59%, respectively. These results confirm the superior ability of PSVK-shFcG-GM/B7.1 over the other constructs in inducing CTL responses specific to the survivin and hCGβ antigens.

### Vaccination with the experimental vaccine can facilitate the release of IFN-γ in mouse

**Figure 4:**
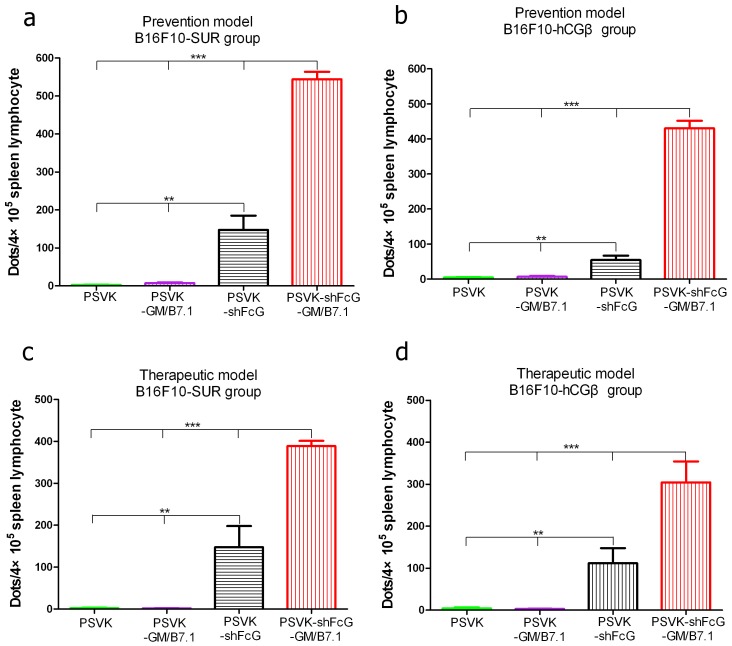
Measurement of IFN-γ secretion using an ELISPOT assay. Splenocytes were collected from each group and were stimulated with human survivin (a.a. 80–88, AYACNTSTL) and human hCGβ (a.a. 109–118, TCDDPRFQDS) synthetic peptides. IFN-γ-producing cells were enumerated using an ELISPOT assay. The results are expressed as the number of spots per 4×105 cells and were analyzed by Student’s t-test. (a) Prevention model, B16F10-SUR group; (b) Prevention model, B16F10-hCGβ group; (c) Therapeutic model, B16F10-SUR group; (d) Therapeutic model,B16F10-hCGβ group. ** Significant differences as indicated by a statistical analysis of p<0.01. *** Significant differences as indicated by a statistical analysis of p<0.001.

Peptide-specific T cells from vaccinated mice were counted using an IFN-γ ELISPOT (both immunization strategies; **Fig. 4a-b**: prevention model; **Fig. 4c-d**: therapeutic model). We found that IFN-γ was increased significantly in the splenocytes of PSVK-shFcG-GM/B7.1 vaccinated mice. The number of spots observed in these groups was higher than that in other groups (P<0.001 by one-way ANOVA), and the PSVK-shFcG vaccinated group had a higher number of spots too. These data indicate that PSVK-shFcG-GM/B7.1 vaccination significantly enhances the release of the cytokine IFN-γ.

### Identification of tumor-infiltrating lymphocytes (TILs)

**Figure 5:**
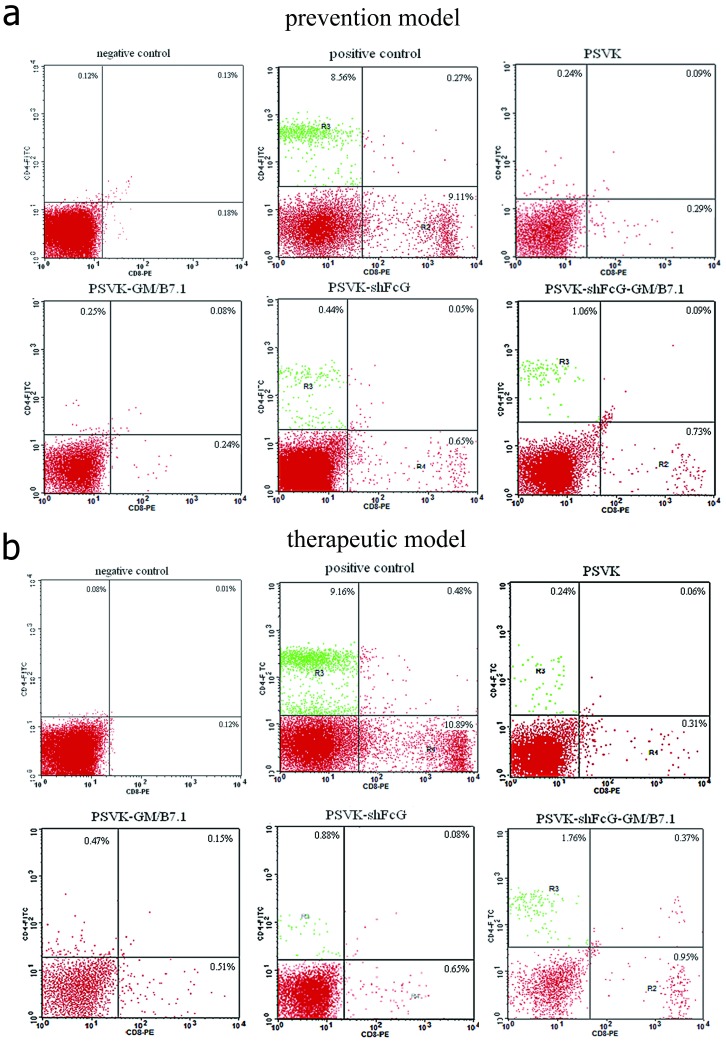
Identification of tumor-infiltrating lymphocytes (TILs). Flow cytometry to determine the percentage of CD4+ and CD8+ T lymphocytes. (a): prevention model; (b): therapeutic model. In the upper row from left to right is negative control, positive control and PSVK group; in the lower row from left to right is: PSVK-GM/B7.1, PSVK-shFcG and PSVK-shFcG-GM/B7.1 group. In these two immunization model, the CD4+ and CD8+ T lymphocytes could be detected in the tumor tissues of mice immunized with plasmids containing the fusion antigen gene fragments PSVK-shFcG-GM/B7.1 and PSVK- shFcG, but the corresponding cells were not detected in the other groups.

CD4^+^ and CD8^+^ T lymphocytes were detected by flow cytometry in the tumor tissues of the mice immunized with the plasmids containing the fusion antigen gene fragments (PSVK-shFcG-GM/B7.1 group and PSVK-shFcG group), but the corresponding cell populations were not detected in the other groups (both immunization strategies; **Fig. 5a**: prevention model; **Fig. 5b**: therapeutic model). (P<0.01,one-way ANOVA). The difference between PSVK-shFcG-GM/B7.1 groups and PSVK-shFcG groups of vaccinated mice was not considered statistically significant. (P > 0.05, Student’s t-test).

## Discussion

The utilization of traditional DNA vaccines is determined by many factors such as the carrier system, the target genes, and the microenvironment. Typical adaptations to augment immunogenicity are performed using multivalent constructions and cocktail administration. The disadvantage of this strategy is a reduced level of gene expression, which induces a weaker immune response. Secondly, there is great concern that the DNA vaccine may integrate into the host cell genome.

Developments in the theory and technology of immunology, molecular biology and cell biology have lead to the emergence of more efficient and safer forms of vaccines. Alphavirus vectors, at present, have frequently been used as vehicles to generate tumor vaccines [36]. This anti-tumor vaccine combines the advantages of traditional DNA vaccines, RNA vaccines and RNA replicon vaccines based on the following. (1) This vaccine has enhanced stability and ease of production, storage, and transportation compared with RNA vaccines and RNA replicon vaccines. (2) The presence of the replicase genes and the increase in mRNA replication will result in higher levels of gene expression compared with traditional DNA vaccines. (3) The self-replication and transcription of this vaccine occurs in the cytoplasm and will eliminate the risk of integration into the host cell genome, thus greatly improving the safety. (4) Because the efficient replication and translation mechanisms consume most of the resources of the host cell, the vaccine will eventually induce the apoptosis of the transfected cells, which could lead to clearance by the body and a reduction in immune tolerance [11], [37], [38], [39]. In summary, this SFV vector-based DNA vaccine can be expected to achieve better immune efficacy and safety and has broad prospects in the development of therapeutic vaccines.

DNA vaccines have the additional advantage of possessing a recombinant construction, which allows the integration of many epitopes by gene fusion. Also it can significantly improve immunity [40], [41], [42], [43], [44]. We utilized the strategy of co-expressing tumor-specific antigen genes and adjuvant genes using a bicistronic plasmid and subsequently evaluated the protective effects induced by these candidate vectors. In this study, we prepared recombinant DNA plasmids, which encode the most prominent cytotoxic T lymphocyte epitopes of human survivin and chorionic gonadotropin β chain-CTP37. Survivin was considered to have a ubiquitous expression in tumor cells and rare expression in normal cells. As a component of hCGβ, CTP37 is a hallmark protein of many tumors and is responsible for tumor metastasis and immunological tolerance. Given the homology of CTP37 between human and monkey (76%), we constructed a chimeric fusion gene in which different domains are derived from human and monkey.

To explore whether the effect of the survivin-CTP37 DNA vaccine can be enhanced by combination with molecular adjuvants, we investigated the effects of the cytokine GM-CSF, B7.1, the IgG Fc fragment and the signal peptide of GPI. Host APCs are a critical factor for the presentation of tumor antigens [45], [46]. However, the maturation and infiltration of APCs can be inhibited by tumors [47], [48], [49]. Because the anchor signal peptide GPI can localize to the cell membrane, the fusion protein will be presented on the cell membrane and recognized by APCs via the APC Fc receptor. Concerning the activation of T cells, it is known that B7.1 is required as a co-stimulatory signaling molecule and that binding of B7.1 to CD28 facilitates adhesiveness between lymphocytes and tumor cells. More importantly, the percentage of B7.1-positive cells was significantly lower in poorly differentiated primary carcinomas and metastatic carcinoma cells. Therefore, to circumvent inadequate B7.1 co-stimulation and to augment the immune response, we incorporated the B7.1 gene into the DNA vaccines. The Fc segment of human IgG1 can bind both human and murine dendritic cells (DCs) efficiently [50]. Based on the characteristics of the GPI signal peptide and the Fc fragment, the fusion protein will be presented on the cell membrane and will be recognized by APCs via the Fc receptor. Consequently, the efficiency of antigen presentation will be improved, and immune tolerance will be circumvented.

Even though the effect of vaccination can be improved when several distinct antigens and cytokines are co-administered with different constructs, the precise temporal and spatial co-delivery of antigen is not achieved. It has been reported that co-expression of cytokine and antigen genes in vivo will provide a more conducive microenvironment for the uptake and presentation of antigen by dendritic cells or macrophages. The temporal and spatial co-delivery of antigens and cytokines are responsible for this advantage. Therefore, using a bicistronic plasmid, we constructed a series of constructs that could co-express survivin-CTP37 or cytokine. Subsequently, we tested the ability of these constructs to induce antitumor immunity in animal models.

As expected, the mice vaccinated with the PSVK-shFcG-GM/B7.1 construct induced the strongest anti-tumor immune response against B16F10-SUR and B16-F10hCGβ cell challenge, using both vaccination strategies. The DNA-based replicon vaccine not only suppressed tumor growth in vivo but also prolonged the survival of the tumor-bearing mice. This protection was determined to be the synergistic effect of humoral and cell-mediated immunity. A high antibody titer was induced, and a strong correlation between CTL activity and the efficiency of protection against tumor cells was observed. The assessment of morphology and immunological mechanisms showed that both immunization strategies tested (the preventive model and treatment model) can be effective in inhibiting tumor growth. To assess cytokine bias and the magnitude of the response in splenocytes, a quantitative ELISPOT analysis was conducted. ELISPOT indicated that IFN-γ was significantly induced in the splenocytes of vaccinated mice after the inoculation of the recombinant DNA constructs, in particular with the chimeric construct. IFN-γ is a proinflammatory cytokine that has multiple effects on the immune system, such as the upregulation of the expression MHCII and costimulatory molecules on professional and nonprofessional antigen-presenting cells (APCs). The increase in IFN-γ may induce chemokine secretion and result in the chemoattraction of other immune cells. Challenging mice with tumor cells before or after vaccination allowed us to assess the therapeutic efficacy of our recombinant constructs. These studies demonstrated that the anti-tumor activities of the constructs were extremely distinct based upon the tumor growth in the mice. Among the tested constructs, the DNA vaccine harboring the comprehensive molecular adjuvants (the gene-vaccine test construct) was very effective against pre-existing tumors compared to the other constructs. The greater effectiveness of the gene-vaccine construct may be ascribed to the functions of GM-CSF, B7.1 and IgG Fc.

In conclusion, vaccination with the DNA-based replicon vaccine plasmid PSVK-shFcG-GM/B7.1, which contained GM-CSF as adjuvants, provides insight into the important immune components of anti-tumor immunity that have a synergistic role in the immune response. The effective immune response elicited by the heterogeneous survivin-CTP37 construct combined with the cytokine adjuvant suggested a promising approach that could be developed in the future to break immune tolerance. Taken together, the data presented here may help to develop therapeutic vaccines for reducing metastasis after tumor surgery.
